# A case of psoriasis and atopic dermatitis-like rash induced by interleukin-17 and interleukin-23 inhibitors successfully controlled with upadacitinib

**DOI:** 10.1016/j.jdcr.2025.06.033

**Published:** 2025-07-05

**Authors:** Toshiki Okazaki, Takehiro Takahashi, Riichiro Sugiura, Ryoko Shimada-Omori, Yoshihide Asano

**Affiliations:** a Department of Dermatology, Tohoku University Graduate School of Medicine, Sendai, Japan; b Department of Dermatology, National Defense Medical College, Saitama, Japan

**Keywords:** AD-like rash, Janus kinase inhibitor, psoriasis, upadacitinib

## Introduction

Psoriasis is an immune-mediated disorder manifesting in the skin, joints, or both.^1^ Biologic treatments are an effective therapeutic option, producing long-term remission of psoriasis with a high safety profile.^1^ However, they are occasionally associated with development of eczema or atopic dermatitis (AD)-like rashes as a secondary skin condition, leading to treatment discontinuation.^2^ While atopic eczema arises from systemic T-helper 2 (Th2)-mediated inflammation, psoriasis is driven by an immune response involving Th17 cells, which secrete high levels of interleukin (IL)-17 and IL-22.^3^ Due to their distinct immune pathways, cases of concomitant psoriasis and atopic eczema are considered rare.^3^ Similarly, several reports have documented psoriasis induction by blocking IL-4/IL-13 signaling pathways, which are key mediators of Th2-mediated diseases.^4^ Here, we report a case of psoriasis who developed AD-like rash with IL-17 and IL-23 inhibitors, experienced exacerbation of psoriatic lesions with IL-4/IL-13 inhibition, and ultimately achieved control of both conditions with a Janus kinase inhibitor (JAKi), upadacitinib.

## Case report

**Fig 1:**
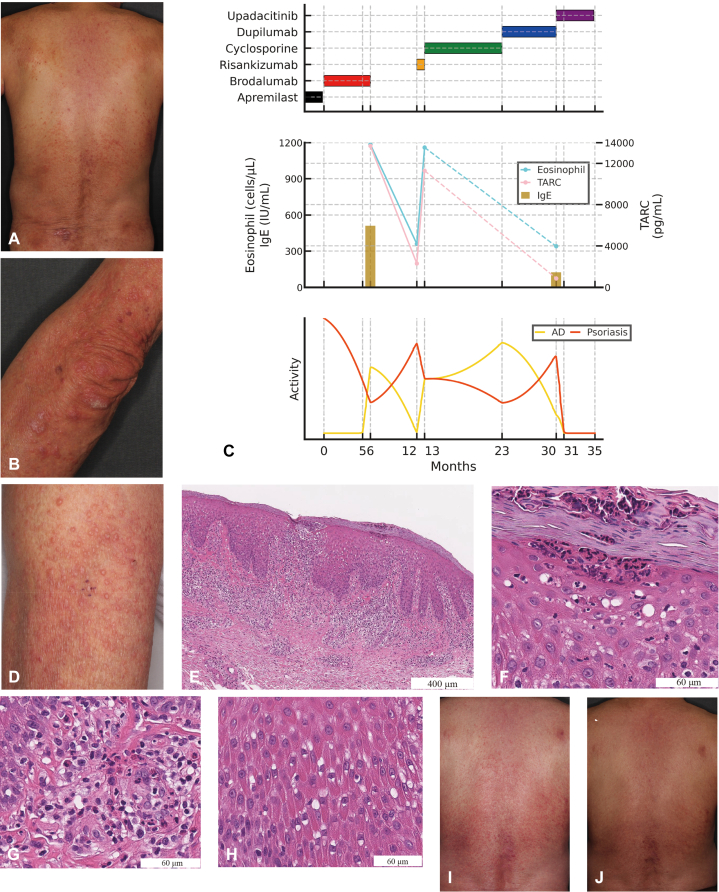
Skin manifestations, histopathological findings, and clinical timeline of the case. **A** and **B,** Physical examination revealed multiple demarcated erythematous plaques partially covered by silvery lamellar scales. **C,** Longitudinal timeline of treatment, laboratory biomarkers, and psoriasis/AD-like rash activity. *Horizontal bars* indicate periods of drug administration: apremilast (*black*), brodalumab (*red*), risankizumab (*orange*), cyclosporine (*green*), dupilumab (*blue*), and upadacitinib (*purple*). Month 0 corresponds to the initiation of brodalumab therapy. Trends in peripheral blood eosinophil count (cells/μL; *cyan line*), serum IgE (IU/mL; *dark goldenrod bars*), and TARC (pg/mL; *pink line*) are shown. Dashed segments connect the 13- and 30-month values for eosinophils and TARC. Smoothed curves represent longitudinal activities of AD-like rashes (*yellow*) and psoriasis (*red*). **D,** Six months after the administration of brodalumab, multiple pruritic erythema and papules appeared, distinct from the typical psoriatic skin manifestation. **E,** Hematoxylin and eosin staining of a skin biopsy specimen from the papule on the forearm demonstrated acanthosis with elongation of rete ridges. **F,** Neutrophil clustering and parakeratosis (Munro's microabscess) were detected. **G** and **H,** Superficial perivascular dermatitis with infiltration of lymphocytes and eosinophils, and epidermal spongiotic vesicles were also detected. **I,** Dupilumab aggravated psoriatic plaques on the back. **J,** One month after the initiation of upadacitinib, skin lesions were almost completely cleared, leaving post-inflammatory pigmentation. *AD*, Atopic dermatitis; *TARC*, Thymus and Activation-Regulated Chemokine.

A 69-year-old Japanese male was referred to our clinic for worsening skin lesions of psoriatic arthritis (PsA). He was diagnosed with PsA 10 years before and had been treated with apremilast, an oral phosphodiesterase 4 inhibitor, for a year. Physical examination revealed multiple demarcated erythematous plaques partially covered by silvery lamellar scales (Fig 1, *A* and *B*) and mild polyarthralgia affecting bilateral fingers. Brodalumab, a human anti-IL-17 receptor A monoclonal antibody, was introduced with the discontinuation of apremilast (clinical course, key lab data, and treatment timeline summarized in Fig 1, *C*). Although the psoriatic lesions temporarily improved, multiple pruritic erythema and papules emerged 6 months after the administration of brodalumab, which were distinct from the typical psoriatic skin manifestation (Fig 1, *D*). His medical history was negative for AD. Laboratory examination revealed marked elevations in Thymus and Activation-Regulated Chemokine (TARC) (13,686 pg/mL), nonspecific IgE (511 IU/mL), and eosinophils (1185/μL). A skin biopsy from a papule on the forearm demonstrated not only classic psoriatic features such as acanthosis with elongation of rete ridges and neutrophil clustering surrounding parakeratosis (Munro’s microabscess), but also eczematous changes including epidermal spongiotic changes and perivascular lymphocytic and eosinophilic infiltration in the papillary dermis, suggesting an overlap of psoriasis and eczema (Fig 1, *E-H*).^5^ Based on the clinical presentation and histopathological findings, brodalumab-induced AD-like rash overlapping with pre-existing psoriasis. Six months after discontinuation of brodalumab, these eczematous lesions improved with a reduction in TARC (2296 pg/mL) and eosinophils (360/μL). On the other hand, due to the exacerbation of psoriasis secondary to the discontinuation of systemic treatment, we decided to use risankizumab, an IL-23 p19 inhibitor (Fig 1, *C*). However, 1 month after the first injection of risankizumab, eczematous reactions developed again, with elevated TARC (11,290 pg/mL) and eosinophils (1160/μL), leading to the discontinuation of risankizumab. We then introduced cyclosporine at 150 mg/day, resulting in only slight improvement. Due to exacerbating eczematous rashes, dupilumab, an anti-IL-4 receptor-α monoclonal antibody, was introduced. While dupilumab alleviated eczematous lesions, it conversely aggravated psoriatic plaques (Fig 1, *C* and *I*). Eventually, we employed upadacitinib, a JAKi 1 approved for both AD and PsA, with the discontinuation of dupilumab. One month after administration of upadacitinib, both psoriatic skin lesions and AD-like rash almost completely cleared (Fig 1, *C* and *J*). There has been no aggravation of either psoriasis or the AD-like rash for 2 years.

## Discussion

Biologic therapy has dramatically improved our ability to treat psoriasis with high therapeutic efficacy and safety.^1^ However, some patients with psoriasis may develop AD-like rash during treatment with biologic agents.^6^ The pathogenesis of these reactions has not been fully elucidated. AD-like eczema during anti-IL-17 inhibition may be explained by the inhibition of Th17, leading to repolarization toward the Th2 arm.^3^ It has been reported that in psoriasis patients, IL-17 inhibitors (secukinumab, ixekizumab, and brodalumab) had adjusted incidence rates of eczematous rashes ranging from 1.11 to 1.83 per 100 000 person-years. By contrast, IL-23 inhibitors (guselkumab, risankizumab, and tildrakizumab) demonstrated lower rates (0.56 per 100 000 person-years) and were associated with a significantly lower risk compared with both IL-17 inhibitors and tumor necrosis factor inhibitors (0.94 per 100 000 person-years).^2^ Indeed, for psoriasis patients with AD-like rash associated with IL-17 inhibitors, switching to IL-23 inhibitors is suggested due to their relatively rare eczematous reactions.^2^ However, in our case, risankizumab also induced eczematous eruptions. The mechanism of this phenotype switching induced by IL-23 blockers has also been hypothesized: IL-23 inhibition leads to tumor necrosis factor inhibition, which in turn results in increased IFN-α production.^7^ IFN-α is considered to cause increased expression of IL-22 receptors in keratinocytes, and IL-22 is suggested as one of the central cytokines in AD.^7^ Similarly, it is known that dupilumab occasionally induces psoriatic manifestations in AD patients, which is considered to be caused by T-cell polarization to Th17 due to the blockade of the Th2 pathway.^4^ After discontinuing dupilumab, we administered upadacitinib focusing on JAK1. It reduces not only Th2 inflammation, by blocking central Th2 cytokines, such as IL-4, IL-13, and IL-31, but also Th1 responses by inhibiting IFN-γ signaling. Moreover, suppression of IL-6 limits the activation of dendritic cells and macrophages and lowers IL-23 secretion, leading to inhibiting Th17 polarization.^8^ Of note, Gargiulo et al reported phenotypic switching from AD to psoriasis during treatment with upadacitinib.^9^ In this respect, further investigation is warranted. Although the histological findings indicated concomitant psoriasis and AD-like change, we cannot entirely exclude the possibility that the patient had a pre-existing overlap of psoriasis and AD. However, in our case, AD-like lesions emerged after brodalumab initiation, which were distinct from the typical psoriatic skin manifestation, accompanied by elevated TARC, IgE, and eosinophils, and resolved after drug withdrawal (Fig 1, *C*). Our histopathological analysis might delineate the sequential histologic features of this phenotypic conversion. As the use of biologics and small molecule inhibitors continues to increase, clinicians are expected to encounter these biologic-induced dermatologic adverse events more frequently. Further elucidation of the underlying mechanisms is necessary, and JAKis may be a viable treatment option in cases where multiple pathophysiological immune pathways are intricately involved, as in our case.

## Conflicts of interest

None disclosed.
